# The microbiome and inborn errors of metabolism: Why we should look carefully at their interplay?

**DOI:** 10.1590/1678-4685-GMB-2017-0235

**Published:** 2018

**Authors:** Karina Colonetti, Luiz Fernando Roesch, Ida Vanessa Doederlein Schwartz

**Affiliations:** ^1^ Programa de Pós-Graduação em Genética e Biologia Molecular, Universidade Federal do Rio Grande do Sul, Porto Alegre, RS, Brazil; ^2^ Laboratory of Basic Research and Advanced Investigations in Neurosciences (BRAIN), Hospital de Clínicas de Porto Alegre, Porto Alegre, RS, Brazil; ^3^ Interdisciplinary Research Center on Biotechnology-CIP-Biotec, Universidade Federal do Pampa, Bagé, RS, Brazil; ^4^ Medical Genetics Service, Hospital de Clínicas de Porto Alegre, Porto Alegre, RS, Brazil

**Keywords:** Inborn errors of metabolism, microbiome, microbiota, diet, treatment

## Abstract

Research into the influence of the microbiome on the human body has been shedding new light on diseases long known to be multifactorial, such as obesity, mood disorders, autism, and inflammatory bowel disease. Although inborn errors of metabolism (IEMs) are monogenic diseases, genotype alone is not enough to explain the wide phenotypic variability observed in patients with these conditions. Genetics and diet exert a strong influence on the microbiome, and diet is used (alone or as an adjuvant) in the treatment of many IEMs. This review will describe how the effects of the microbiome on the host can interfere with IEM phenotypes through interactions with organs such as the liver and brain, two of the structures most commonly affected by IEMs. The relationships between treatment strategies for some IEMs and the microbiome will also be addressed. Studies on the microbiome and its influence in individuals with IEMs are still incipient, but are of the utmost importance to elucidating the phenotypic variety observed in these conditions.

## Introduction

The human body host a large amount of non-human genetic material, the microbiome, defined as the set of microorganisms, their genes, and the surrounding environmental conditions (Marchesi and Ravel, 2015). The human gut microbiome is believed to play an important role in the development of basic physiological systems, such as the digestive, immune, and nervous systems, and constitutes a virtual metabolic organ of unquestionable importance (Lopez-Legarrea *et al.*, 2014; Suez *et al.*, 2014; Maukonen and Saarela, 2015). The gastrointestinal (GI) tract is a metabolically rich environment that harbors approximately three-quarters of the body’s immune cells, contains vagal afferent endings which respond to immune cells and immune and bacterial products (cytokines, proteases, 5-Hydroxytryptimaine and CRH for corticotropin-releasing hotmone, CRH, histamine), and has receptors for compounds produced by neuroendocrine cells (Omran and Aziz, 2014). Diet and genes related to the immune system and metabolism are among the key factors with potential to alter the bacterial community present in the gut. Thus, the associations of diet, metabolism, the central nervous system, and the immune system with the development and composition of the gut microbiome has become the object of intense interest among the scientific community (Mayer *et al.*, 2014).

Inborn errors of metabolism (IEM) are rare monogenic genetic diseases characterized by absent or deficient activity of a given enzyme and which can sometimes be managed with dietary strategies. The phenotypic heterogeneity found in IEMs is manifested mainly by the age at onset of symptoms, presence (or absence) of neurological compromise, and response to the treatment. In untreated phenylketonuria (PKU) and in propionic and methylmalonic acidemia patients, for instance, the neurological and behavior impairment are highly variable. The development of liver disease is common to several IEMs, such as tyrosinemia type 1 and urea cycle disorders. Also, the response to the treatment is not the same among patients with the same genotype.

Convergent efforts of professionals in different fields have enabled the discovery of new mechanisms and processes whereby the microbiome can exert local and systemic effects. In this non-systematic review of the literature, we will focus on how the gut microbiome could influence the context of treatable IEMs.

## The human gut microbiome

Among the various microbial habitats found in the human body, the GI tract harbors the vast majority of microbial cells (Sender *et al.*, 2016). The composition of the microbiota varies along the GI tract, both quantitatively and qualitatively, depending on the environmental conditions (pH, oxygen, etc.) (Donaldson *et al.*, 2016). In the small bowel (particularly the duodenum), the composition is similar to that of the stomach, while the large bowel (especially the colon) contains the majority of the gut’s microbial population, as it is the site of fermentation, due to the availability of nutrients obtained from digestion (Madigan and Martinko, 2006).

Prior to the development of next-generation sequencing (NGS) techniques, the gene profile of these microorganisms had never been determined accurately (Grenham *et al.*, 2011). The ability to obtain a large number of gene sequences in a short period of time and at relatively low cost led to the acquisition of an immense volume of data to which biological significance could then be ascribed (Cho and Blaser, 2012). Advances in these techniques, coupled with the development of bioinformatics tools, have allowed analysis of the gut microbiome to an extent that would have been impossible with bacterial cultures alone (Hiergeist *et al.*, 2015). Furthermore, the use of NGS and bioinformatics techniques, with the aid of databases and computational and statistical algorithms, has allowed complex studies for the detection, quantification, and functional analysis of the human microbiome and its physiological associations, thus expanding knowledge of microbial ecology beyond simple pathogen *vs.* host relationships.

Initiatives such as the Human Microbiome Project, created in the United States in 2008, have sought to characterize the microbial communities of various sites in the human body, with a focus on analyzing the role of these microorganisms in sickness and in health (Human Microbiome Project Consortium, 2012). In Europe, a similar effort known as MetaHIT, which took place from 2008 to 2012, sought to study the association of the gut microbiome with several states of health and illness, prioritizing obesity and inflammatory bowel disease (Metagenomics of the Human Intestinal Tract, MetaHIT.

The results of the aforementioned initiatives have led to a new appreciation for the human microbiome from taxonomic and functional points of view. The microbiota is both functionally relevant and uniquely personal, differing even between monozygotic twins, what suggests that childhood exposure to different environmental factors is a determinant of development of the adult microbiota (Turnbaugh and Gordon, 2009). Despite great interpersonal variation in the microbiota, the metabolic roles of its microorganisms are highly conserved: enriching the biosynthesis of cofactors and vitamins, in addition to a key role in central carbohydrate metabolism, aromatic amino acids (AA), and ATP synthesis in the lower GI tract (Segata *et al.*, 2011; Human Microbiome Project Consortium, 2012). This has given rise to the notion of a “functional core” of microorganisms rather than a core set of microbial taxa, as the same essential roles can be played by different taxa (Lloyd-Price *et al.*, 2016).

The gut microbiota is influenced by the environment and affected by diet, medications, age, geographic factors, surgical interventions, and host genetics, particularly genes related to the immune system and metabolism (Yatsunenko *et al.*, 2012; Dabrowska and Witkiewicz, 2016; Goodrich *et al.*, 2016). The gut microbiome suffers drastic changes during the first three years of life (Yatsunenko *et al.*, 2012). After that, diet is one of the main factors that shape the gut microbiota (De Filippo *et al.*, 2010; David *et al.*, 2014), and the microbiome continues to evolve all lifelong (Ottman *et al.*, 2012; Odamaki *et al.*, 2016). Once diet is strongly correlated with cultural habits and is affected by geographic factors, such as availability of nutrients and source of carbohydrates, fibers and fat, one can also consider that culture affects the patterns found in the microbiome (Yatsunenko *et al.*, 2012). To study the microbiome is also to study ecology. From an ecological point of view, maintaining sufficient bacterial diversity and richness is important for gut microbiota functional redundancy, adaptability and to provide a certain tolerance against environmental challenges, resilience (Gill *et al.*, 2006). Western diets, rich in calories and refined sugar, are associated with lower richness in microbial communities at individual level (alpha diversity) and higher variation among individuals (beta diversity) when compared with diets high in fiber and relatively low in calories (Martínez *et al.*, 2015). Individuals who consume a Western type diet with high-energy and high-fat intake present changes in metabolic and immune biomarkers, such as a higher body mass index and higher levels of inflammatory markers than those who follow a high-fiber, low-calorie diet (Cani *et al.*, 2009). Taken together, these facts have led to associations between microbial richness and health. Once microbial richness is strongly associated with diet patterns (De Filippo *et al.*, 2010; Cotillard *et al.*, 2013; Sonnenburg and Sonnenburg 2014), both the composition and energy content of one’s diet are important modulators of the microbiota (Oriach *et al.*, 2016). Diet is a crucial driver of the composition of the microbial community from childhood to old age (Kashtanova *et al.*, 2016) and has the potential to alter the bacterial metabolite profile, thus influencing the host’s metabolism both directly and indirectly.

The major bacterial metabolites known to influence the host include short-chain fatty acids (SCFAs) and vitamins. SCFAs are organic monocarboxylic acids with six or fewer carbon atoms, generated by anaerobic fermentation of indigestible dietary fibers (such as cellulose, xylans, and inulin) in the gut. The main SCFAs produced as a result of these fermentation processes are butyrate, acetate, and propionate. SCFAs are absorbed by the host and are important energy sources, corresponding to 10% of the energy source in a Western diet. Portal and hepatic veins contain large amounts of SCFAs (Cummings *et al.*, 1987). SCFAs also stimulate growth of bacteria in the genera *Lactobacillus* and *Bifidobacterium*, these playing a key role in colon physiology and metabolism (Roy *et al.*, 2006) and influencing the immune and inflammatory responses (Maslowski and Mackay, 2011; Tremaroli and Bäckhed, 2012; Lopez-Legarrea *et al.*, 2014). *In vitro*, SCFAs increase the production of anti-inflammatory cytokines, such as IL-10, and decrease production of proinflammatory cytokines, such as IL-1β, IL-6, and TNF-α (Vinolo *et al.*, 2011). Production of SCFAs also promotes transcription of the *PTH1* gene, which encodes tryptophan hydroxylase, the rate-limiting enzyme of serotonin synthesis in the gut (Reigstad *et al.*, 2015). SCFAs are also generally involved in G-protein signaling, modulation of cell signaling, cell–cell interactions, gene expression, immune function, and neurotransmitter synthesis and release (Nakao *et al.*, 1998; Le Poul *et al.*, 2003; Nguyen *et al.*, 2007; Han *et al.*, 2014; Nankova *et al.*, 2014). Several physiological effects, including regulation of energy homeostasis, obesity, immune system functions, cancer, and cerebral function, as well as histone deacetylase (HDAC) inhibition, have been associated with butyrate (Koh *et al.*, 2016). Specific host transporters and receptors are available for butyrate, and it is also used by colon cells as a source of energy through beta-oxidation (Stilling *et al.*, 2016). Furthermore, acetate and propionate can be used by the liver for lipogenesis and gluconeogenesis, respectively (Janssen and Kersten, 2015). The potential for modulation of host metabolism and genetics by the gut microbiota suggests that the role of this factor warrants closer attention. This is especially true in IEMs in which metabolic pathways are originally altered, as the microbiome may act to reinforce metabolic pathways that are advantageous or disadvantageous to the host, with a direct impact on phenotype.

The evidence for a role of the composition of the human gut microbiota and its metabolites in health and illness becomes increasingly stronger (Sharon *et al.*, 2014; Coleman and Nunes, 2016; Rooks and Garrett, 2016). Changes in the GI tract microbiota induce metabolic changes with systemic effects (Tremaroli and Bäckhed, 2012; Ochoa-Repáraz and Kasper, 2014; Sharon *et al.*, 2014), and current research seeks to characterize microbiota–host interactions to elucidate the depth and breadth of this influence.

Some conditions, such as liver and bowel diseases and *Clostridium difficile* infection, are already being treated with microbiota-modifying therapies. These include probiotics, prebiotics, antibiotics, and fecal transplant (Sheth *et al.*, 2016; Young, 2017). Probiotics are living microorganisms that, when administered at an appropriate concentration, can confer health benefits to the host, while prebiotics are indigestible components of foods that benefit the host by promoting growth or activity of a specific bacterial species or community in the colon. Fecal transplant is the administration of fecal matter from a healthy donor to a diseased individual, with the objective of restoring the typical microbial community of the healthy gut. These strategies can be used jointly or in isolation to restore the balance of the intestinal microbial community in the event of dysbiosis, which is any change to the composition of resident commensal communities relative to the community found in healthy individuals.

## Inborn Errors of Metabolism (IEM)

IEMs are individually rare diseases, but as a group they are fairly common. Currently, more than 600 known human diseases are classified as IEMs (Alfadhel *et al.*, 2016). Classically, IEMs are defined as a set of monogenic (single-gene) diseases that cause protein dysfunction, with partial or total loss of enzyme activity; however, IEMs can be pleiotropic, and may involve virtually any organ or system. Clinical onset may occur from even before birth up to adulthood (Sharer, 2011), and environmental triggers may be crucial determinants of individual phenotype (Lanpher *et al.*, 2006). In an individual IEM, one primary metabolite flux is affected. In complex disease, however, a whole network of metabolite fluxes might be subtly altered to contribute to the overall phenotype. This concept of metabolic flux is essential in the translation of genetic and environmental factors into the phenotype or threshold for disease (Lanpher *et al.*, 2006). Even a single metabolite defect can affect several secondary metabolic pathways, with a greater or lesser degree of environmental influence, to contribute to each patient’s specific phenotype.

The treatment and management of IEMs are always individualized, based on each patient’s diagnosis and phenotype, and there is broad heterogeneity even within each category (Argmann *et al.*, 2016). Despite this heterogeneity in management approaches, the specific treatment usually falls into one of three classes: (I) enzyme replacement therapy, to replenish the deficient enzyme; (II) substrate reduction therapy; or (III) dietary treatment, although organ transplantation is also used in some cases (Ezgu, 2016). Additional non-specific treatment may be necessary, depending on the presence of comorbidities, such as neuropsychiatric disorders in PKU patients (Bilder *et al.*, 2017), or renal and neurologic impairment in patients with tyrosinemia type I (Santra *et al.*, 2008; Chinsky *et al.*, 2017). Given the importance of diet to the microbiome, we will primarily address dietary therapy in this review, with a secondary focus on the importance of the microbiome in allogeneic hematopoietic stem-cell transplantation (HSCT).

Dietary treatment for IEMs may be employed as monotherapy or adjuvant therapy. Its purpose is to eliminate or reduce whichever toxic compound that accumulates in the body (Schwartz *et al.*, 2008). However, this form of therapy has several limitations, including overload and/or deficiency of certain food groups and nutrients (Crenn and Maillot, 2007; Boyer *et al.*, 2015). Theoretically, diets restricted or excessively rich in certain nutrients may prompt a state of intestinal dysbiosis with systemic effects, leading to malnutrition, obesity (Henao-Mejia *et al.*, 2012), type 1 (Wen *et al.*, 2008) or type 2 diabetes (Larsen *et al.*, 2010), inflammatory bowel disease (Ashton *et al.*, 2017; Geirnaert *et al.*, 2017) and liver disease (Lee and Sokol, 2015), as well as a variety of disorders featuring an inflammatory component, symptoms of autism spectrum disorders (De Angelis *et al.*, 2015), and even cancer (Jacqueline *et al.*, 2017; Xu and Jiang, 2017). Studies seeking to identify the effects of dietary treatment and nutrient supplementation on the microbiome of patients with IEMs are still scarce. A summary of this research will be presented below and in Table 1.

**Table 1:** Inborn errors of metabolism addressed in this review, main phenotypic features, and overview of management.

EIM (Substrate accumulated)	Affected protein/gene	Main clinical features	Long-term management	Reference
Phenylketonuria (Phenylalanine)	Phenylalanine-4-hydroxylase (*PAH*)	Neurologic impairments, with physical, cognitive, and behavioral consequences, even in well-controlled PKU	Restriction of dietary phenylalanine. Phe-free medical formula. Sapropterin (BH_4_) supplementation in responsive patients. Large neutral amino acids (LNAA)	Regier and Greene, 2000; OMIM #261600
Tyrosinemia type I (Tyrosine, maleylacetoacetate, fumarylacetoacetate, and succinylacetone)	Fumarylacetoacetate hydrolase (*FAH*)	Hepatomegaly, acute liver failure, cirrhosis and hepatocellular carcinoma Episodic paralysis and episodic peripheral neuropathy Renal Fanconi syndrome, renal failure, glomerulosclerosis, nephromegaly, nephrocalcinosis Gastrointestinal bleeding, paralytic ileus Pancreatic islet-cell hypertrophy, splenomegaly Rickets, chronic weakness	Dietary management with reduced intake of phenylalanine and tyrosine; Nitisinone Liver transplantation	Das, 2017; Sniderman et al., 2006; OMIM #276700
Urea cycle disorders (Ammonia)	Carbamoylphosphate synthetase I (*CPS1*); Ornithine transcarbamylase deficiency (*OTC*); Argininosuccinate Synthase 1 (*ASS1*); Argininosuccinate lyase (*ASL*), Arginase-1 (*ARG1*); N-acetylglutamate synthase (*NAGS*); Ornithine transporter (*SLC25A15*); or citrin (*SLC25A13*)	Vomiting, lethargy, and behavioral abnormalities. Neurologic impairments. Seizures in acute hyperammonemia. Liver impairments	Dietary management with reduced intake of proteins, Essential amino acids supplementation. Vitamin and mineral supplementation, Medications to increase the nitrogen excretion. Liver transplantation,	Ah Mew et al., 2003; Häberle et al., 2012.
Alkaptonuria (Homogentisic acid and its oxidation products)	Homogentisate 1, 2-dioxygenase (*HGD*)	Urine that turns dark on standing, alkalinization, black ochronotic pigmentation of cartilage and collagenous tissues, arthritis (especially in the spine). Cardiovascular impairments: Aortic and/or mitral valve calcification, coronary artery calcification, aortic dilatation. Urolithiasis, ochronotic prostate stones (in males)	Nitisinone ^*^	Introne and Gahl, 2003; Mistry et al., 2013; OMIM #203500
Propionic acidemia (Propionic acid)	Propionyl-CoA carboxylase (*PCC*)	Central nervous system impairments: acute encephalopathy, lethargy, axial hypotonia, limb hypertonia, coma, seizure, psychomotor retardation, cerebral atrophy, dystonia, cerebellar hemorrhage (rare), ischemic stroke in the basal ganglia (rare). Decreased appetite, feeding difficulties, vomiting, dehydration. Hepatomegaly, pancreatitis. Pancytopenia, neutropenia, anemia, thrombocytopenia. Cardiomyopathy, tachypnea, apnea. Osteoporosis, dermatitis acidemica	L-carnitine, Antibiotics, Low-protein diet, Vitamin and mineral supplementation, Precursor-free amino acid and/or isoleucine/ valine supplementation.	Baumgartner et al., 2014; OMIM #606054https://www.ncbi.nlm.nih.gov/pmc/articles/PMC4180313/
Methylmalonic Acidemia (Methylmalonic acid)	Methylmalonyl-CoA mutase (*MUT*)	Central nervous system impairments: lethargy, hypotonia, developmental delay, coma, severe involvement of globus pallidus, delay in myelination, cerebellar hemorrhage (rare), ischemic stroke in the basal ganglia (rare)}. Leukopenia, thrombocytopenia. Cardiomyopathy, hepatomegaly, pancreatitis, recurrent episodes of vomiting, interstitial nephritis, chronic renal failure	Same as in PA, plus vitamin B12 in responsive patients.	Baumgartner et al., 2014; OMIM #251000
Hemochromatosis type 1 (Iron)	HFE protein, Hemochromatosis gene (*HFE1*)	Heart involvement: cardiomyopathy, congestive heart failure, arrhythmia, cardiomegaly. Liver involvement: cirrhosis, hepatomegaly, hepatocellular carcinoma. Diabetes mellitus. Arthritis. Hypogonadotropic hypogonadism. The severe effects of the disease usually do not appear until after decades of progressive iron loading	Periodic phlebotomy	Seckington and Powell, 2000; OMIM #235200
Trimethylaminuria (Amino-trimethylamine)	Flavin-containing monooxygenase 3 (*FMO3*)	Behavioral/psychiatric manifestations: depression, suicidal, psychosocial problems in school. In some patients: anemia, neutropenia, pulmonary infections; tachycardia and severe hypertension after eating cheese.	Dietary restriction of: Trimethylamine and its precursors including choline and lecithin Trimethylamine N-oxide; Inhibitors of FMO3 enzyme activity, such as indoles. Use of: acid soaps and body lotions, activated charcoal and copper chlorophyllin, antibiotics, riboflavin supplements.	Phillips and Shephard, 2007; OMIM #602079

* Under investigation

Organ transplantation (mainly liver transplantation and HSCT) is also a treatment option for several IEMs (Sirrs *et al.*, 2013; Boelens *et al.*, 2014). Within this context, the microbiome was recently noted as a key factor in graft-*vs.* -host disease (GVHD). Acute GVHD is characterized by rupture of the intestinal barrier, caused by the conditioning regimen administered before HSCT and by leakage of microbe-associated molecular patterns (MAMPs, also known as pathogen-associated molecular patterns or PAMPs), particularly lipopolysaccharide (LPS). The proinflammatory response mounted against these molecules leads to systemic inflammation. Antibiotic treatment in the perioperative period of allogeneic HSCT has been associated with a higher likelihood of GVHD and lower odds of survival, which suggests a potentially pathogenic role of antibiotics through depletion of gut microbiome diversity. The finding that fecal transplant successfully treats GVHD by reconstituting the microbiota has reinforced this theory (Balmer *et al.*, 2014; Melis *et al.*, 2014; Kakihana *et al.*, 2016; Rashidi *et al.*, 2017; Routy *et al.*, 2017; Spindelboeck *et al.*, 2017). Efforts to characterize the influence of the microbiome in complications resulting from organ transplantation are paving the way for new avenues of treatment. Administration of *Lactobacillus*, for instance, appears to be a promising strategy for treatment of GVHD in allogeneic HSCT recipients, although the mechanism of action has yet to be fully understood (Staffas *et al.*, 2017).

## Influence of the microbiome on the major organs affected by IEMs

**Figure 1:**
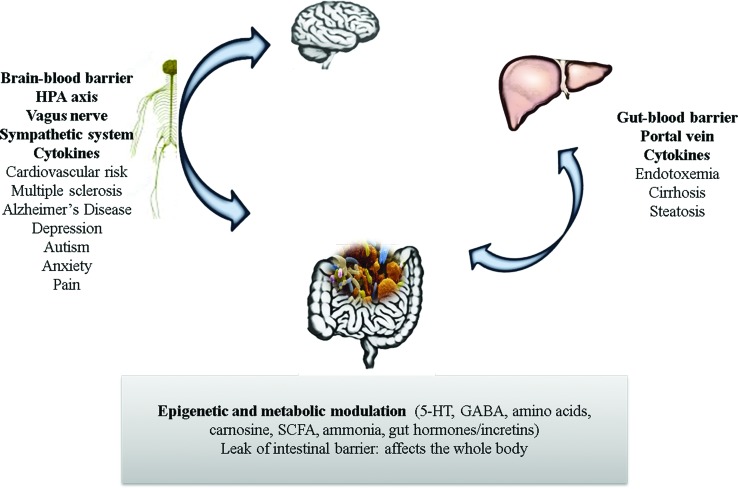
Known effects of the gut microbiome on the main organs affected in an IEM. In bold are the ways by which the interactions occur. Below are the features related to the gut microbiota and the organs. The gut microbiome produces several metabolites and actively participates in the biosynthesis of vitamins and cofactors, metabolism of carbohydrates, proteins and lipids. The gut microbiota interacts with the whole body via the immune and endocrine systems. The two major organs affected in an IEM are the brain and the liver. In addition to the components of the immune and endocrine systems, the described gut-brain interactions also involve the brain-blood barrier, HPA axis, vagus nerve and the sympathetic system. This may predispose to several diseases, such as increased cardiovascular risk, multiple sclerosis, Alzheimer’s disease, depression, autism, anxiety, and also can be related to pain. Interactions with the liver can occur via the portal vein, the gut-blood barrier, and can be involved in several hepatic diseases, most of them linked to endotoxemia.

The features of IEMs are highly heterogeneous; however, the nervous system central (CNS) and liver, due to their high metabolic rate, are particularly susceptible to the effects of any metabolic defect (Sahoo *et al.*, 2012). These organs are also closely related to microbiome activity, and a summary of on this matter can be found in Figure 1.

The microbiome has wide-ranging influence on the CNS, with probable effects on metabolism (Fu *et al.*, 2015; Montagner *et al.*, 2016), coordination (Sampson *et al.*, 2016), mood (Slykerman *et al.*, 2017), behavior (Tillisch *et al.*, 2013), cognition (Steenbergen *et al.*, 2015), temperature control (Chevalier *et al.*, 2015), and sensation (Chiu *et al.*, 2013). This influence may begin before birth, via the maternal microbiome (Rautava *et al.*, 2012), and may be perpetuated throughout life, playing essential roles in the development of the blood–brain barrier (Braniste *et al.*, 2014), maturation of the immune system (Chung *et al.*, 2012), and also myelination of the prefrontal cortex (Hoban *et al.*, 2016). Communication between the microbiome and the CNS is two-way, occurring both through metabolites and toxins produced by the bacterial community on the one hand, and via the immune, metabolic, nervous, and endocrine systems on the other (Powell *et al.*, 2017). Over the years, disruption of the microbiome-brain-gut axis has been associated with various diseases. A breach in system homeostasis may occur at any point along this axis. Stressful situations affecting the brain, for instance, may affect the gut microbiome via the hypothalamic-pituitary-adrenal (HPA) axis, with repercussions for immune cell activity and bowel function (Moloney *et al.*, 2014). Bacterial components, in turn, can stimulate secretion of proinflammatory cytokines from epithelial cells, dendritic cells, and macrophages. Knowingly, several neuropsychiatric disorders, including depression, anxiety, schizophrenia, and autism spectrum disorders, are associated with elevated circulating levels of proinflammatory cytokines (Liu *et al.*, 2015a; Petra *et al.*, 2015). In addition to these pathways, cerebral function can also be modulated by microbial metabolites capable of crossing the blood–brain barrier (Li and Zhou, 2016). Pierre and Pellerin (2005) reported that monocarboxylate transporters (MCTs), which transport lactate, pyruvate, ketone bodies, and other SCFAs, are widely expressed in cerebral tissue, and especially so in the cortex, hippocampus, striatum, and cerebellum (Pierre and Pellerin, 2005). In rats, G protein-coupled receptors (GPCRs) activated by propionic acid (PPA) are also highly expressed in brain tissue (Bonini *et al.*, 1997). Antibiotic therapy, which is commonly used in the treatment of some IEMs, depletes the microbiome and can affect levels of neuromodulatory substances (tryptophan, monoamines, and neuropeptides), thus influencing anxiety and cognition patterns (Desbonnet *et al.*, 2015).

As evidence mounts for a systemic effect of the gut microbiome on the host, the liver has also been found to be affected by changes in the microbiome. In addition to its central role in intermediary metabolism (for instance, many enzymes affected by IEM are only expressed in liver) and bile secretion, the liver is the target organ of therapies for metabolic disorders (Brunetti-Pierri and Lee, 2005) and can also be considered a secondary lymphoid organ (Macpherson *et al.*, 2016). Changes in liver physiology are probably caused primarily by DNA methylation processes, covalent histone modifications, and regulation of gene expression by non-coding RNA (*nc*RNA) (Macpherson *et al.*, 2016). In addition to SCFAs, isothiocyanates and polyphenols are also produced by the microbiome, and all of these compounds have the potential to cause epigenetic changes. As the liver receives blood from the gut through the portal vein, it is susceptible to exposure to microbial byproducts that cross the intestinal barrier. In humans and non-human animals alike, whenever liver or bowel disease causes dysfunction of the barrier role played by these organs, there is a breakdown in mutualism between the host and the microbiome, which leads to systemic exposure to gut bacteria and increased immune activation (Chassaing *et al.*, 2015). In these situations, the liver becomes a primary immune barrier that mediates host–microbiome mutualism (Balmer *et al.*, 2014).

Hepatocytes are sensitive to microbial byproducts, and may trigger an inflammatory immune response with systemic effects: even exposure to low levels of LPS induces IFN-γ overexpression and IL-10 underexpression in the liver in animal models of obesity, thus predisposing to the development of steatohepatitis (Yang *et al.*, 1997). On the other hand, deletion of the flagellin receptor TLR5 in mouse hepatocytes has been shown to predispose to hepatic steatosis and fibrosis, as well as other features of the metabolic syndrome. In this study, antibiotic treatment was able to reverse steatosis and related aspects in TLR5 knockout mice, suggesting that mechanisms for clearance of microorganisms capable of gut–liver translocation is essential for maintenance of host systemic health, preventing the chronic inflammation induced by microbial pathogens (Etienne-Mesmin *et al.*, 2016). Taking into account the important immune role of the liver, it makes sense that most patients with cirrhosis and severe liver failure die of sepsis, not of metabolic derangements (Leber *et al.*, 2009), as many of these infections are caused by oral commensals or gut microbiota (Gustot *et al.*, 2009). The dysbiosis state itself impulses inflammatory response and has potential for causing disease. The role of the microbiome in liver disorders is further supported by the efficiency of treating these conditions with probiotics, prebiotics, and antibiotics. Studying the microbiome, hence, may provide a better understanding of complex diseases and lay the groundwork for new therapies (Tilg *et al.*, 2016).

## The microbiome and IEMs: the state of the art

The gut microbiome plays roles in amino acid and carbohydrate metabolism, vitamin and cofactor biosynthesis, and production of SCFAs, in addition to influencing the physiology of the liver, brain, and GI tract, all of which are affected by IEMs. In light of the many important activities of this virtual metabolic organ and its vast impact on the host, some studies have considered the microbiome as a factor that interferes with organic homeostasis in the context of IEMs, and have sought to characterize possible interactions, both endogenous (genetic defect) and exogenous (treatment/diet), with host metabolic pathways, as well as the probable consequences of the presence or absence of specific bacteria and their metabolites on the human body.

**Figure 2:**
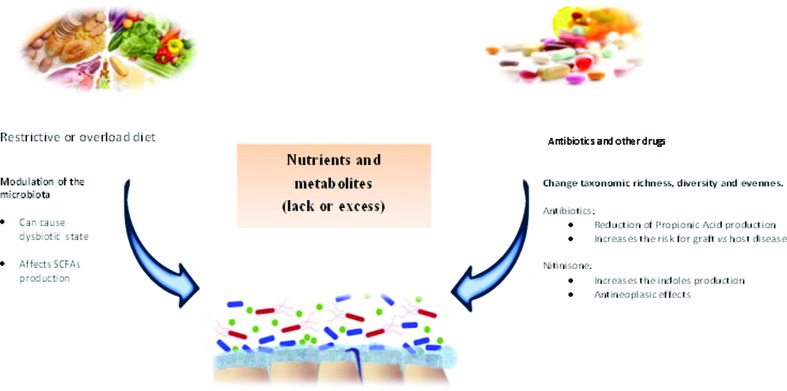
Common treatments used in IEM and its effects over the microbiome. Diet is an important modulator of the microbiome, and also is a very common treatment for several IEMs. Diets with restriction or abundance of certain nutrients can cause a dysbiotic state, leading to an abnormal immune signaling (inflammation), leaking of gut-blood barrier, and breaking of the energetic balance of cells, with potential to affect the whole body. Antibiotics, on other hand, cause rapid and significant drops in taxonomic richness, diversity and evenness. This can bring benefits, as in the case of propionic/methylmalonic acidemia patients, by decreasing the levels of propionic/methylmalonic acid, or not, as in the case of organ transplants, once patients treated with antibiotics during the perioperative period had an increased risk for graft *vs.* host disease. Organ transplantation is a treatment for several IEMs. Other drugs used for treating this class of genetic disease can also affect the microbiome or metabolite production, like nitinisone, used in treatment of tyrosinemia type I, that raises the levels of indoles which in turn have antineoplasic effects.

Studies of the association between microbiome and IEMs have focused on aminoacidopathies (such as PKU, tyrosinemia, and alkaptonuria), organic acidemias (methylmalonic acidemia and propionic acidemia), and hemochromatosis. The main characteristics of the IEMs addressed in these studies, including their long-term management, are summarized in Table 2. Some possible effects of treatments of IEM on microbiome are showed in Figure 2.

**Table 2:** Summary of experimental studies addressing the role of the microbiome in inborn errors of metabolism.

Reference	EIM	Model	Experimental design	Aims	Findings
Ney et al., 2017	Phenylketonuria	Human	Randomized, controlled, crossover trial; Early-treated PKU subjects consumed, for 3-wk. each, their usual low-Phe diet combined with AA-Formula or GMP; Metabolomics analysis of a subset of plasma and 24-h urine samples; Dietary intake.	To assess metabolites and neurotransmitters derived from Tyr and Trp in plasma and urine samples from subjects with PKU consuming both AA-formula and GMP	Plasma metabolome: 7 of the 40 microbiome-associated compounds showed differential levels with AA-formula compared with GMP; Significant differences in the plasma profile of secondary bile acids; Associated compounds showed differential levels with AA-Formula compared with GMP; Urine metabolome: 7 of 45 microbiome; Individuals fed with AA formula had a 50% higher intake of Tyr and Trp; Differential degradation level of Tyr by intestinal microbes of individuals fed with AA-formula, potential harmful metabolites formed; Higher metabolism of Trp via the kynurenine pathway might be linked with inflammation patterns;
					Reinforces prebiotic properties of GMP.
Pinheiro de Oliveira et al., 2016	Phenylketonuria	Human	Observational, cross-sectional study, convenience sampling strategy; Review of medical records for Plasma Phe and Tyr levels, and daily Phe intake; Questionnaire including questions on comorbidities, use of medicines, and dietary intake; V4-16S rRNA gene sequencing; Metagenome prediction.	To characterize the microbiome of PKU patients	Decreased levels of Families *Clostridiaceae*, *Erysipelotrichaceae*, and *Lachnospiraceae*, class *Clostridiales*, genera *Coprococcus*, *Dorea*, *Lachnospira*, *Odoribacter*, *Ruminococcus*, and *Veillonella*.
MacDonald et al., 2011	Phenylketonuria	Human	8-week open-label, single-arm, pilot intervention; Infants aged between 4 weeks and 6 months; Formula Phe-free with prebiotic to replace a regular infant formula phe -free without prebiotics; Measurement of Phe levels in blood; Record of stool frequency, size, appearance, and consistency; Stool samples analyzed for pH and bacterial groups (Fluorescence in situ hybridization technique).	Influence of prebiotic scGOS/lcFOS addition to an infant Phe-free protein substitute	Bifidobacteria and lactobacilli–enterococci levels were similar to those of healthy breast-fed infants and greater than those reported for infants on infant formula without prebiotics.
Sawin et al., 2015	Phenylketonuria	Mice C57BL6/J PKU (Pah^enu2^)	PKU (Pah^enu2^) and wild-type mice were fed with isoenergetic (Aminoacid, GMP, or casein) diets for 8 week; Three experiments were done; Measurement of SCFA by gas chromatography; Quantification of plasma cytokines; Analysis of splenocyte T cell populations by flow cytometry.	Prebiotic effects of GMP	Increased SCFAs levels; Decreased levels of inflammatory cytokines Decreased quantity of the Proteobacteria, genus *Desulfovibrio*.
Durrer et al., 2017	Phenylketonuria	Mice C57BL6/J (PAH^enu2^ mutant)	*In vitro* and *in vivo* test of a probiotic expressing the phenylalanine lyase gene; Measurement of phe plasma pre and post-treatment; Measurement of enzyme activity; Prebiotic mixed into chow; Fecal culture and immunogenic evaluation.	Assessment of a genetically engineered probiotic (GMO)	Reduction of plasma Phe levels in the mouse model of PKU; Survival of GMO Lactobacillus reuteri 100-23C in the mouse gastrointestinal tract, but no permanent colonization; No immune response to transgenic protein.
Gertsman et al., 2015	Alkaptonuria Tyrosinemia	Human	Collection of samples from patients with alkaptonuria before and after treatment with NTBC plus samples of Tyrosinemia types I, I and transient patients; Analysis of the sera y tQ-TOF LC/ MS metabolomic platform; Untargeted metabolomics strategy; *In vitro* experiments with cultures of human cells and intestinal flora cultures to identify the nature of the link between 4-HPP and the elevated indoles.	Evaluate the metabolic effects of nitisinone	Increased levels of I3CHO, in patients treated with nitinisone.
Frye et al., 2016	Propionic acidemia (PA)	Human lymphoblastoid cell lines (LCLs)	Measurement of mitochondrial function in ASD and sex-age-matched control LCLs; Incubation with PPA and reactive oxygen species.	Effects of PPA in an unfavorable redox microenvironment	PPA can have both beneficial and toxic effects on mitochondrial function, depending on concentration, exposure duration, and microenvironment redox state.
Buhnik-Rosenblau et al., 2012	Hemochromatosis type 1	Mouse	Comparison between wild-type and genetically deficient mouse; Culture followed by tRFLP and 16S rRNA gene sequencing.	Effects of iron metabolism (Irp2^-/-^ and Hfe^-/-^ genes) on microbiome	Irp2^-/-^ Increased levels of *L. intestinalis* compared to Hfe^-/-^ mice and *L. murinus* compared to both Hfe^-/-^and WT mice; Hfe^-/-^ Increased levels of *Enterococcus faecium*; Increased levels of *L. johnsonii* to both Hfe^-/-^ and Irp2^-/-^ mice compared to WT.

PKU: phenylketonúria; Phe: phenylananine; Tyr: tyrosine; Trp: tryptophan; scGOS/lcFOS: neutral short chain galactooligosaccharides and long chain fructooligosaccharides; AA: aminoacids; GMP: glycromacropepide; SCFAs: short chain fatty Acids; GMO: genetically modified organism; I3CHO: indole-3-carboxaldehyde (exclusively produced by microbiota); PA: propionic academia; LCLs: Human lymphoblastoid cell lines; PPA: propionic acid; Irp2: iron regulatory protein 2 gene; Hfe: hemochromatosis protein gene. *The identified compounds are either exclusively synthesized or contributed by intestinal bacteria, as well as by human metabolism.

The majority of studies on microbiome–IEM interactions has focused on PKU. One of the most thorough among such studies compared the microbiome of eight patients with PKU to that of 10 healthy individuals by analysis of the 16S rRNA gene. In this study, Pinheiro de Oliveira *et al.* (2016) demonstrated reduced abundance of bacteria in the families Clostridiaceae, Erysipelotrichaceae, and Lachnospiraceae, class Clostridiales, and genera *Coprococcus*, *Dorea*, *Lachnospira*, *Odoribacter*, *Ruminococcus,* and *Veillonella* in patients with PKU, as well as an increase in *Prevotella*, *Akkermansia*, and Peptostreptococcaceae populations. Their metabolic prediction was associated both with starch and glucose metabolism and with AA metabolism (Pinheiro de Oliveira *et al.*, 2016). The authors raised the hypothesis that bacterial enrichment related to LPS biosynthesis, as observed in patients with PKU, might be associated with peripheral inflammation, as indicated by the proinflammatory circulating cytokine profile of these patients (Coakley *et al.*, 2014). In the same study, the authors found a correlation between microbiotic profile and circulating levels of phenylalanine (Phe), which might indicate a relationship between these patients’ microbiome, their treatment response, and their phenotype.

Focusing on the potential impacts of prebiotic treatment in individuals with PKU, a study reported by MacDonald *et al.* (2011) analyzed the effects of prebiotic oligosaccharides (scGOS/lcFOS) as an adjunct to the metabolic formula that forms the mainstay of PKU management. As breastfeeding is highly restricted in children with PKU, the authors theorized that a lack of the oligosaccharides present in breast milk might be associated with increased fecal pH and reduced bifidobacterial populations, thus predisposing the patient to infections. Administration of probiotics might mitigate this problem. The experiment assessed the dominant bacterial groups and found that the administered prebiotic oligosaccharides were able to maintain bifidobacteria levels and low fecal pH, without altering circulating levels of Phe. Despite the small sample size and lack of statistical power, these findings suggest that supplementing metabolic formula with prebiotics might be an interesting strategy in PKU, as the levels of Bifidobacteria and Lactobacilli–Enterococci at the end of the study were similar to those found in healthy children and higher than those reported in children who took the formula alone, without prebiotics. In the only patient who was previously receiving a diet without prebiotics, there was also a reduction in pathogens such as *C. perfringens* and *C. difficile* (group *Clostridium histolyticum/lituseburense*), *E. coli, Shigella, Salmonella*, and *Klebsiella* (subgroup Enterobacteriaceae) (MacDonald *et al.*, 2011).

Also regarding prebiotics, recent years have been promising in terms of the use of glycomacropeptide (GMP) as a substitute for Phe-free AA formula in patients with PKU. GMP is highly glycosylated and, when pure, constitutes a natural protein source that lacks the AAs (Phe, tyrosine (Tyr), tryptophan (Trp), histidine, cysteine, arginine) involved in some IEMs, including PKU (Neelima *et al.*, 2013). For now, human trials are seeking to ascertain the efficiency of GMP as a partial (50% formula, 50% GMP) or total replacement for the Phe-free AA formula. In trials, the use of GMP had no significant impact on circulating Phe levels and was preferred by patients over the formula, as GMP is more palatable and, according to patients, provides greater satiety than a formula-based diet alone (Ney *et al.*, 2016; Zaki *et al.*, 2016). This could make GMP an option to increase treatment adherence.

When the urine and plasma metabolome of the individuals with PKU were compared within the groups fed with AA-formula or GMP, differences were found between the metabolite profile linked to the microbes. There were no differences between fasting plasma concentrations of the Tyr and Trp, but individuals fed with AA formula had a 50% higher intake of Tyr and Trp. This can be explained as a result of higher degradation by the intestinal microbes, raising the levels of microbiome-derived compounds from Tyr. Some of these compounds are potentially harmful. There was no differential degradation of Trp, but the metabolism of Trp via the kynurenine pathway was evidenced by higher levels of metabolites linked to this pathway and might be linked with inflammation patterns. Change in plasma profile of secondary bile acids, but not primary bile acids, supports the statement that there are alterations in the gut microbiome with ingestion of AA-formula and GMP, and reinforces the prebiotic proprieties of the GMP (Ney *et al.*, 2017).

Although the effect of GMP on the human gut microbiome has yet to be studied, in mice, GMP was associated with control of Th2-type immune responses, increased *Lactobacillus* and *Bifidobacterium* populations in as little as three days after treatment (Jiménez *et al.*, 2016), elevated levels of SCFAs and reduced levels of proinflammatory cytokines, and reduced Proteobacteria counts (genus *Desulfovibrio*) without affecting circulating Phe levels (Sawin *et al.*, 2015). The genus *Desulfovibrio* is associated with production of hydrogen sulfate, a cytotoxic compound found at higher levels in patients with ulcerative colitis (Rowan *et al.*, 2010).

Regarding disorders of tyrosine metabolism, Gertsman *et al.* (2015) described the metabolic effect of nitisinone (NTBC or 2-(2-nitro-4-fluoromethylbenzoyl)-1,3-cyclohexanedione) in patients with alkaptonuria. Analysis of their metabolic profile showed that indole levels were increased in treated patients as compared with controls. Indoles play a key role in signaling pathways (as building blocks for melanin and serotonin) and intercellular communication, facilitate quorum sensing, and have been uniquely associated with dietary intake and microbial metabolism of tryptophan. Among the indoles found to be increased, indole-3-carboxaldehyde (I3CHO) is produced exclusively by the microbiota, while the other two are produced by human cells (Gertsman *et al.*, 2015). The authors stressed that the reduced form of I3CHO, indole-3-carbinol, a compound also found in cruciferous vegetables, is associated with the prevention of several neoplasms.

Animal experiments also suggest that genetic defects in the host may alter the composition of the gut microbiota, leading to dysbiosis due to a buildup of substances in the cells or lumen of the bowel (Buhnik-Rosenblau *et al.*, 2012). This effect has been observed in hemochromatosis. Hemochromatosis is a disease caused by excess iron absorption by gut cells, which leads to iron overload. This usually becomes clinically detectable in adulthood and is damaging to many organs, including the liver, pancreas (causing diabetes), heart, and skin (Babitt and Lin, 2011). Mutations in the *HFE* gene account for the majority of cases of hereditary hemochromatosis, especially in individuals of Northern European descent (Barton, 2013). In a study of mice with mutations in two genes that encode proteins involved in regulation of iron homeostasis (*HFE* ^*-/-*^and *Irp2* ^-/-^), Buhnik-Rosenblau *et al.* (2012) found abnormalities particularly in resident populations of lactic-acid bacteria, both in *Irp2-*mutant and in *HFE*-mutant mice as compared to controls.

The gut microbiome produces several metabolites, including PPA, a SCFA implicated in several diseases. In autistic populations, the level of the phylum *Firmicutes* is increased and was largely attributable to *Clostridia* class with *Ruminococcaceae* and *Lachnospiraceae* families. The differences in Clostridia species in children with autism spectrum disorder include greater abundance of *Clostridium* clusters I, II, XI and *C. bolteae* (Finegold *et al.*, 2002; Song *et al.*, 2004; Parracho *et al.*, 2005; Williams *et al.*, 2011; Strati *et al.*, 2017). Several *Ruminococcaceae* and *Lachnospiraceae* are known butyrate producers and may thus influence SCFA levels (Louis *et al.*, 2010). So, the treatment with antibiotics can affect producers of SCFA. Some patients’ symptoms improve transiently when antibiotics are administered (Sandler *et al.*, 2000; Shaw 2010). Curiously, a similar effect is seen in patients with propionic acidemia, who can experience the same neurodevelopmental complications seen in autism (Witters *et al.*, 2016). Among the various roles played by PPA, it was recently reported to act as a modulator of mitochondrial function. In a study of autism and control cell lines, the effects of PPA depended not only on the concentration of the acid, but also on the level of reactive oxygen species (ROS) present, as ROS influence mitochondrial ability to use PPA as an energy source. Thus, PPA could have beneficial effects in individuals without mitochondrial dysfunction, and harmful effects in individuals with an unfavorable metabolic status and elevated levels of ROS (Frye *et al.*, 2016). In methylmalonic acidemia, which shares several symptoms and management strategies with propionic acidemia, vitamin B_12_ (cobalamin) is also used as treatment in responsive patients, in addition to antibiotics. This vitamin is synthesized by some gut bacteria, and is also a regulator of microbiome composition and function (Baumgartner *et al.*, 2014; Degnan *et al.*, 2014).

The microbiome can also be considered an exogenous source of tetrahydrobiopterin (BH_4_), another important metabolite of gut bacteria. BH_4_ is a key cofactor for several regulatory enzymes, as Phenylalanine-4-hydroxylase, which catalyzes the conversion of L-phenylalanine to L-tyrosine. The BH_4_ has also been shown to improve working memory and cerebral activation (Christ *et al.*, 2013). In rodents, BH_4_ production is age-dependent and is related to the presence of Actinobacteria in the bowel, especially *Adlercreutzia equolifaciens* and *Microbacterium schleiferi*. These same species have been identified in the human gut microbiome (Belik *et al.*, 2017). Very little is known about the determinants of responsiveness to BH_4_ therapy and its effects on cerebral activity and cognition, but these effects are known to be multifactorial, as they vary across individuals with the same genotype (Pérez *et al.*, 2005). The discovery that BH_4_ is naturally produced by gut microbiota has implications for translational medicine, as this cofactor is used in the treatment of some patients with PKU.

The long-term perspective is that elucidation of the metabolic role of the microbiota and identification of which species play these roles will pave the way for manipulating the microbiome, so that pathways beneficial to the host are stimulated, while those harmful to the host are inhibited. In this line, some authors have raised the hypothesis of using methanogenic bacteria normally present in the human bowel to control metabolites such as trimethylamine (TMA), bypassing the normal route of trimethylamine N-oxide (TMAO) production as an intermediate for CH_4_ to an alternative pathway (Brugère *et al.*, 2014). In the liver, deficiency in the pathway of TMA conversion into TMAO leads to trimethylaminuria, an IEM that causes strong body odor, impairing the patients’ quality of life and interpersonal relations (Mackay *et al.*, 2011). Diets rich in compounds such as phosphatidylcholine, choline, betaine, and L-carnitine generate TMA via the gut microbiota, which is then converted in TMAO by the liver. High levels of TMAO are associated with increased risk of cardiovascular disease in the general population (Wang *et al.*, 2011; Koeth *et al.*, 2013; Gregory *et al.*, 2015; Liu *et al.*, 2015b). Making the transition from theory into practice, administration of the probiotic *Lactobacillus reuteri,* engineered to express a phenylalanine lyase gene from the cyanobacteria *Anabaena variabilis*, successfully treated mice with PKU. Blood levels of Phe declined after the fourth day of treatment and remained low throughout the experiment, with no permanent colonization of the gut (Durrer *et al.*, 2017), suggesting potential for modified probiotics in the treatment of IEMs.

The creation of genetically modified probiotics design especially to normalize defective metabolic pathways in the host is only one of the many potential advantages of microbiome research. IEMs are characterized by substantial variability in presentation, and genotype alone cannot explain patients’ clinical pictures. The microbiome may contribute significantly to factors such as tolerance to certain nutrients and responsiveness to cofactors (and to treatment itself). Studying the microbiomes of patients with IEMs may provide valuable tools for clinical practice, both advancing our understanding of phenotypes and facilitating the development of new biomarkers and therapies.

## Main questions about microbioma and IEM and how to address them

There are some important issues involved in the study of the human microbiome in IEM. First of all, most of the diseases that compound the IEM class are rare, and usually there are subclasses within the same IEM. This is the reason why the studies normally have a small number of participants. Second, the microbiome is mainly influenced by diet, and diet overload or restriction is one of most common treatments for IEM. This is one of reasons that make obtaining an adequate control group very difficult. Third, this class of diseases is derived of a metabolic genetic defect, and defects in a metabolic gene also affect the microbiome. So, if a dysbiotic state is observed in this group of patients will it reflect the genetic or the diet effect? Taken together, all the facts above make it very hard to obtain a homogeneous and statistically valid group of untreated patients and make difficult the comparison pre and post-treatment to verify if the altered microbiome is mainly affected by genetic or diet effects. Additional difficulty is added by the fact that several metabolic diseases, if untreated, can lead to severe impacts through life, so IEM patients should start to be treated as soon as possible.

Despite the difficulties, studying the patterns of the microbiome in groups of treated patients offers the possibility to evaluate the real impact of the genetic defect and diet on the microbiome. Patients need lifelong treatment, and the intragroup study of phenotype, microbiome and diet can be elucidative for some ancient questions that remain unknown. PKU patients, for instance, were studied in light of the microbiome by Pinheiro de Oliveira *et al.* (2016) (see Table 2). Even though not capable of answering the question if alteration comes from diet or genetics, a microbiome alteration correlated with Phe blood levels was observed. This is exciting data, due to the fact that it can help explain why some patients are more tolerant to Phe than others, despite having the same genotypes.

In an IEM, the genetic defect and the diet factors coexist, so the measure of macro- and micronutrients ingested is required. Diet has a strong impact on the microbiome, and in spite of patients having similar lines of treatment all over the world, the source of fibers, carbohydrates and proteins can vary geographically and/or culturally. For this reason, microbiome studies should not combine patients of geographically distinct regions or culture to raise the number of participants. Rather, these studies must be done locally and then, if methodologically possible, make comparisons that take into account the dietetic/cultural/geographic factors.

As detailed above, there are several other factors that can influence and be influenced by the microbiome. Important data as sex, age, body mass index, type of birth delivery, breast feeding (duration and transition to solid food), antibiotic and other drug usage, vitamin supplementation, as well as physical exercise, and other diseases (physical and/or mental) must be collected and also analyzed. All subjects included in studies that aim to characterize the microbiome of certain IEMs should be three years or older to avoid the period of drastic changes in microbiome composition due to the typical change in diet during this period. Given that the microbiome varies according to the stage of life and sex, and certain cultures can also exert some influence, the best way to avoid interference of age and sex is the sex-age-matched strategy.

Another useful strategy is based on experimental studies using animal models. This strategy is very important since animal models have less genetic variation and are maintained in a highly controlled environment (that includes diet and/or a germ-free environment). Also, a high the number of subjects can be easily obtained in such research. This is the better model for initial tests of genetically engineered probiotics and correlations with diseases caused by the genetic defect in the absence or presence of the treatment. This kind of study, besides not being capable of fully reproducing the human reality, can work to generate hypotheses and help to provide better strategies and comprehension of studies done in humans.

With the development of NGS tools, procedures are no longer the main limitation for human microbiome studies. Microbiome data is currently obtained by three different approaches: 1) by 16S rRNA gene partial sequencing, 2) by whole DNA shotgun metagenomic sequencing, or 3) by metatranscriptomics (mRNA-seq), to access the active gene expression pattern For instance, the 16S rRNA gene sequencing method is largely used and has been the first choice method among researchers. Reasons for choosing this approach include the availability of a comprehensive database and scalability. Moreover, studies based in metatranscriptomics require a better control for sample collection to RNA/metabolites processing. Metagenomics, metatranscriptomics and all other “omics”, and the associated bioinformatics techniques are allowing comparative analyses in an unprecedented way. All of these tools allow for testing a recent hypothesis related to the presence of a common set of microbial taxa universally present in healthy individuals (Turnbaugh *et al.*, 2007), also known as microbial core. However large variations in the taxonomic composition observed in the human microbiome rapidly refute such a hypothesis (Bäckhed *et al.*, 2012). Due to the well-known microbial functional redundancy in nature, an alternative hypothesis is the presence of a functional core represented by a set of metabolic functions that are performed by the microbiome within a particular habitat, but are not necessarily provided by the same organisms in different people (Shafquat *et al.*, 2014). Still, studies devoted to better understand how deeply the microbiome can affect an organism with critical metabolic pathways that are naturally altered, are just in the early stages. Multidisciplinary efforts need to be done to aggregate modern techniques of sequencing and identification of metabolites that can lead to the phenotype or drug effect in question. Microbial sequencing alone will not be capable of explaining the phenotype, but is a fundamental tool in the understanding of the process. Additional techniques based on metabolomics analysis and RNA-seq, as well as gathering information about the immune system and SCFA levels can offer fundamental pieces of information in the process.

## Conclusions

Studies on the microbiome in IEMs are scarce. The effects of the genetic defect itself and of treatment in IEMs, especially in the long term, have yet to be fully understood. As IEMs are commonly managed through dietary intervention (nutrient overload and/or restriction), dysbiosis is a possibility. This dysbiotic status would alter the patients’ already compromised metabolic state even further, inducing or worsening abnormalities in secondary metabolic pathways, and thus contributing to phenotypic manifestations, especially liver and brain involvement. Dysbiosis can be treated with antibiotic therapy, dietary prebiotics, or fecal transplant, alone or in combination. The administration of probiotics engineered to at least partly meet the metabolic needs of the IEM-affected host has practically unexplored therapeutic potential and may constitute an intervention that is simple to administer, yet has a major impact on the patients’ lives. Collectively, microbiome research in patients with IEMs can not only contribute significantly to our understanding of the pathophysiology of these diseases and to the development of new biomarkers and therapies, but also help to improve the long-term quality of life in affected patients.
